# An improved typhoon simulation method based on Latin hypercube sampling method

**DOI:** 10.1038/s41598-022-13151-y

**Published:** 2022-06-03

**Authors:** Jian-peng Sun, Guan-jun Lv, Xiao-gang Ma

**Affiliations:** School of Civil Engineering, Xi’an University of Architecture and Technology, Xi‘an, 710055 China

**Keywords:** Natural hazards, Engineering

## Abstract

In order to further improve the prediction accuracy of typhoon simulation method for extreme wind speed in typhoon prone areas, an improved typhoon simulation method is proposed by introducing the Latin hypercube sampling method into the traditional typhoon simulation method. In this paper, the improved typhoon simulation method is first given a detailed introduction. Then, this method is applied to the prediction of extreme wind speeds under various return periods in Hong Kong. To validate this method, two aspects of analysis is carried out, including correlation analysis among typhoon key parameters and prediction of extreme wind speeds under various return periods. The results show that the correlation coefficients among typhoon key parameters can be maintained satisfactorily with this improved typhoon simulation method. The results show that the improved typhoon simulation method can generate the correlations among all typhoon key parameters satisfactorily. Compared with the traditional typhoon simulation method, the improved typhoon simulation method has higher accuracy in predicting the typhoon extreme wind speed in Hong Kong, increasing by about 8% and 11% respectively at 200 m height and gradient height.

## Introduction

Typhoon is the most often extreme weather on earth and gives a massive threat to the safety of wind-sensitive structures, such as tall buildings, long-span bridges and other unique buildings^1^. The southeast coastal region of China is the typhoon-prone region of the Northwest Pacific area^2^. Thus, to ensure the safety of wind -sensitive structures in these areas, it is essential to determine extreme wind speeds of structures for a given return period which can provide support for the structure design and safety assessment^3,4^.

In past years, many researchers have carried out a series of studies on the prediction of typhoon extreme wind speed. The typhoon simulation method has been developed gradually and widely used to predict typhoon extreme wind speed^5–10^. Within this method, historical typhoon wind data is first used directly or indirectly to determine the probability distributions of typhoon key parameters. Then, a series of typhoon key parameters are generated by Monte Carlo simulation method, and these typhoon key parameters are substituted into the typhoon model for a series of typhoon simulation. Finally, extreme wind speed analysis is used to obtain the extreme wind speeds under different return periods in the specific areas. This method was first proposed by Russell^9^ and applied to the prediction of extreme wind speeds in Texas coast. Subsequently, Batt et al.^5^, Shapiro^11^ and Georgiou et al.^6^ also predicted extreme wind speeds with the typhoon simulation method. But different typhoon models developed by themselves were used in these studies. Later, Vickery and Twisdale^10^ obtained typhoon extreme wind speeds under different return periods along the typhoon-prone coastline of the United States by incorporating its developed typhoon model and filling model into typhoon simulation method. Ishihara and Yamaguchi^12^ obtained extreme wind speeds over complex terrain with the typhoon simulation method and measure-correlate-predict method. Kim and Lee^13^ developed a typhoon simulation method based on models for genesis, intensity, tracks and wind field to estimate the extreme wind speed of future typhoons.

Although the above research improves the application of typhoon simulation method in extreme wind speed prediction by developing typhoon model, increasing sample length and other related aspects, continuous efforts are still required to establish more accuracy typhoon simulation method. To cater for this need, this paper presents an improved typhoon simulation method by incorporating Latin hypercube sampling method into the traditional typhoon simulation method. The proposed method is able to reproduce the correlation among typhoon key parameters accurately and thus can improve the prediction accuracy of extreme wind speed in the typhoon-prone regions. The proposed method is utilized to the prediction of extreme wind speed in different periods in Hong Kong. The effectiveness of this method is verified by comparing the historical typhoon wind data, the improved typhoon simulation method and the traditional typhoon simulation method.

## Improve typhoon simulation method

The steps for the improved typhoon simulation method are summarized as follows.

### Generating the simulated typhoon key parameters with Latin hypercube sampling method

#### Obtaining historical independent parameters with Cholesky decomposition method

Typhoon key parameters including the central pressure difference ∆*p*_*0*_, the translation velocity *c*, the moving direction *θ*, the minimum of closest distance *d*_min_ and the radius to maximum winds *r*_m_ obtained from the historical typhoon wind data are expressed by a vector {*x*}, as follows:

1 $$ \{ x\}^{T} = \left\{ {ln\left( {\Delta p_{0} } \right) \, ln\left( {r_{max} } \right) \, c \, \theta \, d_{min} } \right\} $$

Then, matrix [*S*] and [*R*] can be calculated according to Eqs. (2) and (3). They represent the correlation matrix and covariance matrix of typhoon key parameters respectively, and then the upper triangular matrix [*T*] can be obtained from [*R*] by Cholesky decomposition as shown in Eq. (4).

2 $$ \left[ {S - \lambda_{k} E} \right]\left\{ {\varphi_{k} } \right\} = 0{\kern 1pt} {\kern 1pt} {\kern 1pt} {\kern 1pt} {\kern 1pt} {\kern 1pt} {\kern 1pt} {\kern 1pt} k = 1 \ldots 5 $$

3 $$ \left[ R \right] = E\left\{ {\left[ {x_{i} - E\left( {x_{i} } \right)} \right]{\kern 1pt} {\kern 1pt} {\kern 1pt} {\kern 1pt} \left[ {x_{j} - E\left( {x_{j} } \right)} \right]} \right\}{\kern 1pt} {\kern 1pt} {\kern 1pt} {\kern 1pt} {\kern 1pt} {\kern 1pt} {\kern 1pt} {\kern 1pt} {\kern 1pt} {\kern 1pt} {\kern 1pt} {\kern 1pt} {\kern 1pt} {\kern 1pt} {\kern 1pt} {\kern 1pt} \left( {i,j = 1 \ldots 5} \right) $$

4 $$ \left[ R \right] = \left[ T \right]\left[ T \right]^{^{\prime}} $$

The independent historical key parameter vector {Z} can be calculated from the lower triangular matrix [T]^−1^ (the inverse of the upper triangular matrix [T]) and the typhoon key parameter vector{*x*}, as shown in Eq. (5).

5 $$ \left\{ z \right\} = \left[ T \right]^{ - 1} \left\{ x \right\} $$

#### Generating the simulated independent parameters with Latin hypercube sampling method

In vector {z}, the independent typhoon key parameters represented by each column of data correspond to vector {x}. The central pressure difference ∆*p*_*0*_ and t the radius to maximum winds *r*_m_ can be fitted by lognormal distribution, and the translation velocity *c*, moving direction *θ* and the minimum of closest distance *d*_min_ can be fitted by normal distribution. Based on theory of Latin hypercube sampling method, each curve of probability function corresponding with typhoon key parameters are divided into n equaling intervals (*n* is the typhoon simulation numbers) and *n* random values for *n* equaling intervals can be generated^14^. Then, the vector $\left\{ {z^{\prime}} \right\}$ for the simulated independent parameter can be generated with inverse transformation of all probability functions for historical independent parameters.

#### Generating the simulated typhoon key parameters

In order to obtain the simulated typhoon key parameter vector $\left\{ {x^{\prime}} \right\}$, the upper triangular matrix [*T*] can be multiplied by the above vector $\left\{ {z^{\prime}} \right\}$, expressed as follows:

6 $$ \left\{ {x^{\prime}} \right\} = \left[ T \right]\left\{ {z^{\prime}} \right\} $$

Unlike the Monte Carlo simulation method^7^, by using Latin hypercube sampling method, the correlations among typhoon key parameters can be maintained satisfactorily in generating the simulated typhoon key parameters.

### Generating typhoons with typhoon model

In order to obtain an initial typhoon position, we bring the simulated typhoon key parameters into the typhoon wind field model. Then, it is assumed that the typhoon moves in a straight path, and the moving speed *c* is always maintained until it disappears. The model proposed by Vickery and Twisdale^10^ was used to calculate the central differential pressure ∆*p*_*0*_, and the central differential pressure ∆*p*_*0*_ remains unchanged. This study adopts the empirical typhoon model proposed by Ishihara et al.^15^, which is described in detail in Huang et al.^16^, but I will not elaborate on it here.

### Performing extreme wind speed analysis

This paper uses the typhoon extreme wind speed theory^17^ to calculate the typhoon extreme wind speed.

Assuming that the probability that the wind speed of a typhoon is less than a specific wind speed *v* is *F*_*v*_, if the maximum score of a typhoon is *U*, the probability that the *U* in *n* typhoons is less than *v* is recorded as

7 $$ F\left( {\left. {U < v} \right|n} \right) = \left( {F_{v} } \right)^{n} = F_{v}^{n} $$

In order to calculate the probability that *U* < *v* in *τ* years, Eq. (7) can be transformed into:

8 $$ F\left( {U < v,\tau } \right) = \sum\limits_{n = 0}^{\infty } {F\left( {\left. {U < v} \right|n} \right)} p\left( {n,\tau } \right) $$

It is generally considered that *p*(*n*, *τ*) satisfies the Poisson distribution, and the Eq. (8) is transformed into:

9 $$ F\left( {U < v,\tau } \right) = \sum\limits_{n = 0}^{\infty } {F_{v}^{n} \frac{{\left( {\lambda \tau } \right)^{n} e^{ - \lambda \tau } }}{n!} = } e^{ - \lambda \tau } \sum\limits_{n = 0}^{\infty } {\frac{{\left( {\lambda \tau F_{v} } \right)^{n} }}{n!} = } e^{{ - \lambda \tau \left( {1 - F_{v} } \right)}} $$

where λ is the annual incidence of typhoons within 250 km of the research site. If *τ* = 1, Eq. (9) becomes:

10 $$ F\left( {U < v} \right) = \sum\limits_{n = 0}^{\infty } {F_{v}^{n} \frac{{\left( \lambda \right)^{n} e^{ - \lambda } }}{n!} = } e^{ - \lambda } \sum\limits_{n = 0}^{\infty } {\frac{{\left( {\lambda F_{v} } \right)^{n} }}{n!} = } e^{{ - \lambda \left( {1 - F_{v} } \right)}} $$

It expresses the probability that *U* < *v* in one year.

Record the maximum wind speed of m typhoons obtained by simulation from small to large as *v*_1_, *v*_*2*_,…*v*_*m*_. Then the probability of *U* < *v*_*i*_ is

11 $$ F_{{v_{i} }} = \frac{i}{m + 1}\;\;\;\;\left( {i = 1, \cdots ,m} \right) $$

Combine Eq. (10) to get the probability of *U* < *v*_*i*_ in a year

12 $$ F\left( {U < v_{i} } \right) = e^{{ - \lambda \left( {1 - \frac{i}{m + 1}} \right)}} $$

Extreme wind speeds in typhoons are usually fitted with one of the family of the generalized Extreme Value Distributions. The Type I Extreme Value (Gumbel) Distribution is used in this study and can be written in the form:

13 $$ F(v) = \exp \left[ { - \exp \left( { - \alpha \left( {v - \mu } \right)} \right)} \right] $$

where *v* is the annual maximum wind speed; *α* and *μ* represent the dispersion and mode respectively, which can be calculated by Eq. (12).

## Case study

Hong Kong is used as the concerned area for the prediction of extreme wind speeds in this study.

### Historical typhoon wind data

This article uses the typhoon historical data from 1949 to 2019 observed by the Shanghai Typhoon Research Institute of the China Meteorological Administration to select the typhoon data 250 km away from Hong Kong to obtain the key parameters of the typhoon.

### Correlation coefficients among typhoon key parameters

Xiao et al.^4^ found that there is a certain correlation between the different typhoon key parameters. Therefore, this study conducted a correlation analysis on the key parameters of historical typhoons observed in Hong Kong, and the results are shown in Table 1. Maximum correlation lies between central pressure difference ∆*p*_*0*_ and radius to maximum wind *r*_*max*_ with correlation coefficient − 0.52. Therefore, It is important to simulate a better typhoon key parameter correlation to simulate a better typhoon wind field.

**Table 1:** Correlation coefficients among typhoon key parameters for historical typhoon wind data.

	ln (∆*p*_*0*_)	ln (*r*_*max*_)	*c*	*θ*	*d*_*min*_
ln (∆*p*_*0*_)	1.00	− 0.52	0.22	− 0.13	0.07
ln (*r*_*max*_)	− 0.52	1.00	− 0.10	0.11	− 0.11
*c*	0.22	− 0.10	1.00	− 0.22	− 0.09
*θ*	− 0.13	0.11	− 0.22	1.00	− 0.38
*d*_*min*_	0.07	− 0.11	− 0.09	− 0.38	1.00

By performing procedures for generating the simulated typhoon key parameters with Latin hypercube sampling method as shown in section 2.1, thousands of simulated typhoon key parameters for Hong Kong can be generated and they can be used in the correlation analysis. Through the correlation analysis of the key parameters of the typhoon obtained by the simulation, the correlation coefficients are obtained, as shown in Table 2. The maximum difference of correlation coefficient for typhoon key parameters between improved typhoon simulation method and historical typhoon wind data is only 0.03. The corresponding relative error of correlation coefficient is only about 2%. Simultaneously, by using the traditional typhoon simulation method, thousands of simulated typhoon key parameters for traditional typhoon simulation method are also be obtained and analyzed. The results are shown in Table 3. The maximum difference of correlation coefficient for typhoon key parameters obtained from the traditional typhoon simulation method and historical typhoon wind data is 1.33. The corresponding relative error of the correlation coefficient is about 102%. Therefore, the above analysis results show that correlations among typhoon key parameters can be maintained satisfactorily with an improved typhoon simulation method, and the same result cannot be obtained with the traditional typhoon simulation method.

**Table 2:** Correlation coefficients among typhoon key parameters for improved typhoon simulation method.

	ln (∆*p*_*0*_)	ln (*r*_*max*_)	*c*	*θ*	*d*_*min*_
ln (∆*p*_*0*_)	1.00	− 0.53	0.22	− 0.11	0.05
ln (*r*_*max*_)	− 0.53	1.00	− 0.12	0.10	− 0.10
*c*	0.22	− 0.12	1.00	− 0.19	− 0.10
*θ*	− 0.11	0.10	− 0.19	1.00	− 0.38
*d*_*min*_	0.05	− 0.10	− 0.10	− 0.38	1.00

**Table 3:** Correlation coefficients among typhoon key parameters for traditional typhoon simulation method.

	ln (∆*p*_*0*_)	ln (*r*_*max*_)	*c*	*θ*	*d*_*min*_
ln (∆*p*_*0*_)	1.00	0.01	0.03	− 0.13	− 0.30
ln (*r*_*max*_)	0.01	1.00	− 0.01	− 0.01	0.00
*c*	0.03	− 0.01	1.00	− 0.00	− 0.01
*θ*	− 0.13	− 0.01	− 0.00	1.00	0.95
*d*_*min*_	− 0.30	0.00	− 0.01	0.95	1.00

### Prediction of extreme wind speeds

**Figure 1:**
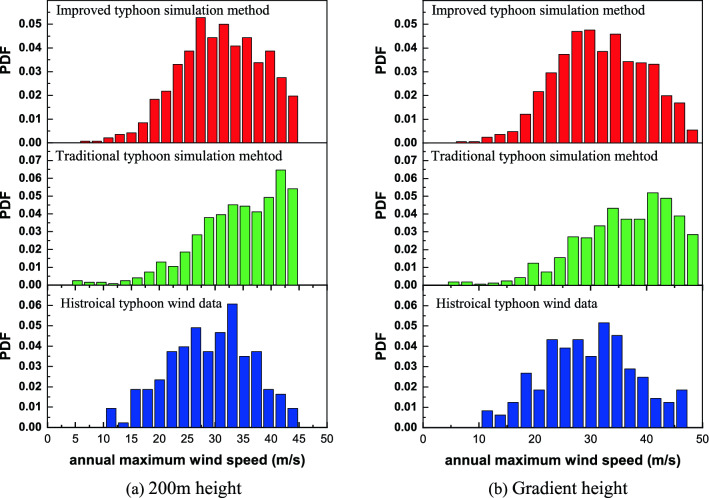
Comparison of probability distribution of annual maximum wind speed.

The maximum annual wind speed sequence of the typhoon at 200 m height and gradient height are important for the simulation of typhoon wind field. This paper uses the key parameters of the simulated typhoon in the previous section, combined with the typhoon model and the typhoon annual incidence *λ* to simulate the maximum annual wind speed sequence and show in Fig. 1. Simultaneously, the maximum annual wind speed sequence at 200 m and gradient heights for improved typhoon simulation method and traditional typhoon simulation method are also be obtained and shown in Fig. 1. Obviously, when the wind speed is less than 32 m/s, the simulation result of the improved typhoon simulation method is greater than that of the traditional typhoon simulation method. On the contrary, in the high wind speed range (> 32 m/s), the simulation result of the improved typhoon simulation method is smaller than that of the traditional typhoon simulation method. In addition, compared with t the simulation result of the traditional simulation method, the simulation result of the improved typhoon simulation method is closer to the simulation result of historical typhoon wind data. The simulation results of the improved typhoon simulation method are similar to the historical typhoon score data, and the probability of occurrence in the range of wind speed 25–35 m/s is larger, and the probability of occurrence in other wind speed ranges is smaller, but the traditional typhoon simulation method has a higher probability of occurrence in the range of wind speed of 40–45 m/s. It is not difficult to infer that when traditional typhoon simulation methods simulate wind fields, many typhoon wind fields will be seriously overestimated and this will reduce the reliability of typhoon extreme wind speed prediction.

**Figure 2:**
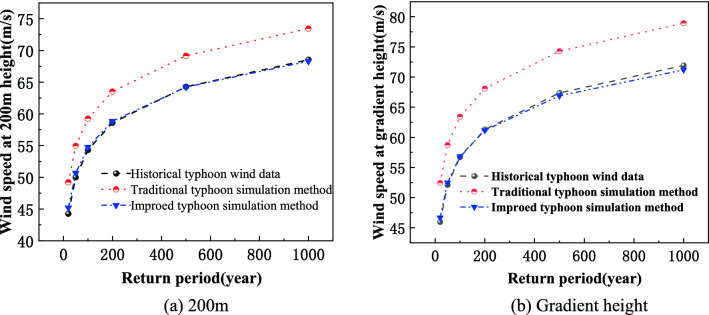
Comparison of typhoon extreme wind speeds under different return periods.

Based on annual maximum wind speed, the Gumbel distribution in conjunction with the extreme wind speed analysis method shown in Sect. 1.3 is used to obtained typhoon extreme wind speed. By using three different typhoon simulation methods (improved typhoon simulation method, traditional typhoon simulation method and historical typhoon wind data), the typhoon extreme wind speeds under different return periods are calculated and shown in Table 4. Comparisons of typhoon extreme wind speeds under different return periods are shown in Fig. 2. It can be known from Table 4 and Fig. 2 that the maximum difference of typhoon extreme wind speeds for all return periods between improved typhoon simulation method and historical typhoon wind data at 200 m height and gradient height are 0.9 m/s and 0.7 m/s, respectively. The maximum difference of typhoon extreme wind speeds for all return periods between the traditional typhoon simulation method and historical typhoon wind data at 200 m height and gradient height are 4.9 m/s and 7.0 m/s, respectively. Compared with traditional typhoon simulation method, the prediction accuracy of typhoon extreme wind speeds obtained with the improved typhoon simulation method can increase about 8%. In general, the simulation results of the improved typhoon simulation method are close to the historical typhoon wind speed data, while the traditional typhoon simulation method and the historical typhoon wind speed data are compared with the historical typhoon wind speed data, and the overall trend is that the maximum extreme wind speed difference is greater in the reproduction period. Therefore, the improved typhoon simulation method will show greater advantages in addition to ensuring the simulation accuracy under the low reproduction period. Therefore, the improved typhoon simulation method used in extreme wind speed analysis can improve the prediction accuracy of typhoon extreme wind speed.

**Table 4:** Typhoon extreme wind speeds under different return periods in Hong Kong (m/s).

Type	Height (3)	Return period (year)					
		20	50	100	200	500	1000
Historical typhoon wind data	500	46.0	52.1	56.7	61.3	67.4	71.9
	200	44.3	50.0	54.3	58.6	64.3	68.5
Improved typhoon simulation method	500	46.7	52.5	56.8	61.2	66.9	71.2
	200	45.2	50.7	54.8	58.8	64.2	68.3
Traditional typhoon simulation method	500	52.4	58.7	63.4	68.1	74.3	78.9
	200	49.2	54.9	59.2	63.5	69.2	73.4

## Conclusion

An improved typhoon simulation method was proposed by introducing Latin hypercube sampling method into traditional typhoon simulation method. This method includes generating the simulated typhoon key parameters with Latin hypercube sampling method, generating typhoon wind field with typhoon model and performing extreme wind speed analysis. Then, this method was validated through correlation analysis among typhoon key parameters and prediction of typhoon extreme wind speed in Hong Kong. The results show that the improved typhoon simulation method can generate the correlations among all typhoon key parameters satisfactorily. The maximum difference of correlation coefficient for typhoon key parameters obtained from the traditional typhoon simulation method and historical typhoon wind data is 1.33. The corresponding relative error of the correlation coefficient is about 102%. Compared with the traditional typhoon simulation method, the improved typhoon simulation method has higher accuracy in predicting height and gradient height. Thus, the improved typhoon simulation method has better simulation results for the extreme wind speed of the typhoon.
